# Superhydrophilic Functionalization of Microfiltration Ceramic Membranes Enables Separation of Hydrocarbons from Frac and Produced Water

**DOI:** 10.1038/s41598-017-12499-w

**Published:** 2017-09-25

**Authors:** Samuel J. Maguire-Boyle, Joseph E. Huseman, Thomas J. Ainscough, Darren L. Oatley-Radcliffe, Abdullah A. Alabdulkarem, Sattam Fahad Al-Mojil, Andrew R. Barron

**Affiliations:** 1 Department of Chemistry, Rice University, Houston, Texas 77007 USA; 2 Energy Safety Research Institute, Swansea University, Bay Campus, Swansea, SA1 8EN Wales UK; 3 Mechanical Engineering Department, College of Engineering, King Saud University, Riyadh, 11421 Saudi Arabia; 4 Department of Civil Engineering, College of Engineering, King Saud University, Riyadh, 11421 Saudi Arabia; 5 Department of Materials Science and Nanoengineering, Rice University, Houston, Texas 77007 USA

## Abstract

The environmental impact of shale oil and gas production by hydraulic fracturing (fracking) is of increasing concern. The biggest potential source of environmental contamination is flowback and produced water, which is highly contaminated with hydrocarbons, bacteria and particulates, meaning that traditional membranes are readily fouled. We show the chemical functionalisation of alumina ceramic microfiltration membranes (0.22 μm pore size) with cysteic acid creates a superhydrophilic surface, allowing for separation of hydrocarbons from frac and produced waters without fouling. The single pass rejection coefficients was >90% for all samples. The separation of hydrocarbons from water when the former have hydrodynamic diameters smaller than the pore size of the membrane is due to the zwitter ionically charged superhydrophilic pore surface. Membrane fouling is essentially eliminated, while a specific flux is obtained at a lower pressure (<2 bar) than that required achieving the same flux for the untreated membrane (4–8 bar).

## Introduction

Although the long-term solution to global energy needs must be based on renewable sources, the present demand for oil and gas shows no sign of a reduction. Unfortunately this comes at a price in terms of resource consumption and possible environmental hazards^1–5^. The introduction of horizontal drilling coupled with hydraulic fracturing or ‘fracking’ has increased the cost of effective access to oil and gas resources, but water usage and wastewater remains controversial issues^6,7^. Hydraulic fracturing uses on average 20 million litres of water per well, of which only 10–15% is typically recovered during the flowback stage^8^. Flowback water as well as post-well completion water (production or produced water) are contaminated with hydrocarbons^9^, many of which are classified as hazardous, which along with significant bacteriological content means that this water cannot be reused without significant treatment^10^. The recovery of clean water for recycling and reuse has been viewed in the past by industry as being economically untenable as produced water is notoriously difficult to purify. As a consequence in most cases the waste water is either evaporated or disposed of through deep injection into abandoned gas or oil wells^11^. In either case storage and transportation of enormous volumes is required, with the potential risk of spillage. Since fresh water is a valuable commodity, wastage is not acceptable, especially in regions of continued drought. Thus, the recyclability of frac (and produced) water is a desirable goal from the environmental and economic viewpoint^12^.

Recyclability of many industrial wastes has been undertaken using ceramic filtration membranes, primarily for their robust nature and the ability to have select pore sizes with narrow distributions. Unfortunately, their capacity to purify or otherwise separate material has many drawbacks, such as membrane fouling leading to low permeate flux^13^. These drawbacks have precluded treatment of flowback and produced water because of submicrion particles, colloids, and hydrophobic organics^14^. Oil emulsions are generally removed by particle filtration (10–1000 μm pore size) and certainly by microfiltration (0.1–10 μm pore size). Shale gas produced water contains 1,000–50,000 mg/L of hydrocarbons, which are divided into the saturate, aromatic, resin and asphaltene (SARA) groupings^9^. While the emulsions represent a challenge with regard to fouling^14^, the solubilized SARA chemicals pose an issue for separation since their molecular weights are much lower than the cut-off for microfiltration. Use of a microfiltration membrane is FDA approved for bacteria removal from water, but hydrocarbons (even high molecular weights) pass through such membranes. Natural organic matter is generally in the 0.001–0.1 μm size, which requires ultrafiltration (0.005–1.0 μm pore size) or nanofiltration (0.0005–0.005 μm pore size). Produced water contains natural organic matter, as well as sugars derived from guar gum (used in frac fluids)^15,16^. If cost and energy consumption were not an issue, then the removal of these impurities may be possible using a multi-stage process to remove each component. It has been suggested that membrane distillation (MD) is an attractive treatment option for shale gas produced water because of its ability to handle high salinity as well as the inherent geothermal heat available to this process^17,18^.

What is needed is a process that removes hydrocarbons and resins at the same time as bacteria and particulates. Due to fouling ultrafiltration and nanofiltration are not suitable^14^. The interaction between membrane surfaces and solutes plays an important role in determining the extent of membrane fouling, explained by the mechanisms of pore blocking, cake formation or hydrophobic interaction^19,20^. Hydrophobic interaction between solutes and membrane material is frequently accepted as one of the predominant mechanisms. Membrane fouling is expected to be less severe with hydrophilic than hydrophobic membranes^21–23^. In addition, the high permeate flux for aqueous eluants is superior to hydrophobic membranes^24^.

**Figure 1:**
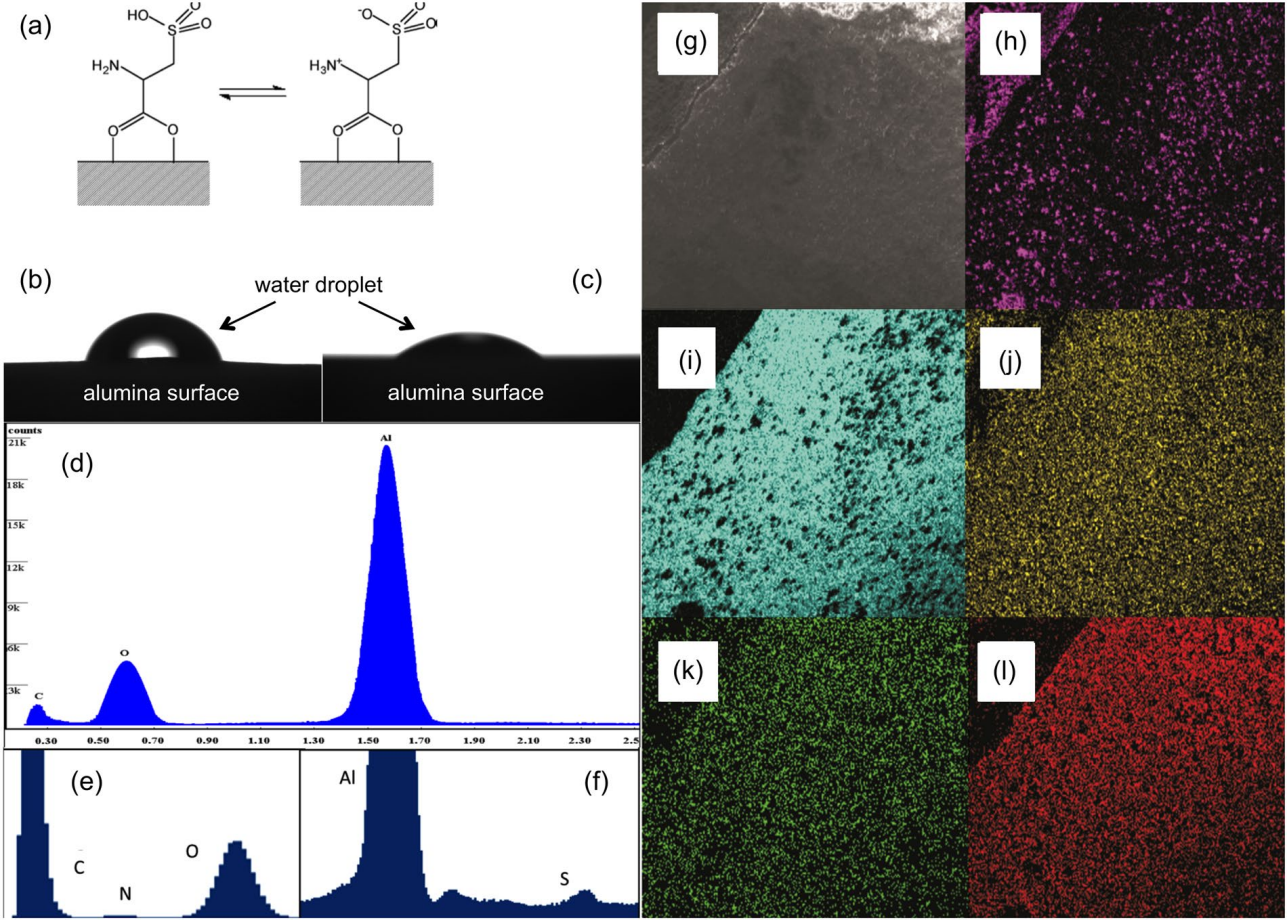
(**a**) Schematic representation of the cysteic acid functionalized alumina surface. Photographic image of a water droplet on (**b**) unfunctionalized alumina and (**c**) cysteic acid functionalized alumina surface taken immediately upon dropping on the surface since within a few seconds the droplet completely wets the surface. EDS of (**d**) as received alumina membrane and cysteic acid functionalized alumina membrane magnified on (**e**) the carbon nitrogen and oxygen peaks and (**f**) on the aluminium and sulfur peaks. SEM image (**g**) of cysteic acid functionalized membrane, with associated EDS maps of cysteic acid functionalized membrane of (**h**) aluminium, (**i**) oxygen, (**j**) sulfur, (**k**) nitrogen, and (**l**) carbon.

There have been various approaches to alter ceramic membranes to be more hydrophilic with antifouling properties: coatings^25^, graft polymerization^26^, and metal substitution^27,28^. However, the chemistry of alumina microfiltration membranes offers the ability to create direct functionalization of the surface (using carboxylic acids) without changing pore size or membrane stability^29^. Furthermore, the use of different functional groups on alumina surfaces allows for changes in the wettability of the surface^30,31^. Cysteic acid (HO_2_CCH(NH_2_)CH_2_SO_3_H) functionalization (Fig. 1a) forms a super hydrophilic surface (contact angle of 5°, see Fig. 1b,c)^32^. Functionalization of the surface of a ceramic membrane also controls the flux rate through the membrane: generally, the more hydrophilic a surface the higher the flux^32^. Our goal is to show that functionalization of an alumina ceramic membrane with cysteic acid should increase the flux through the membrane for a particular pore size and that cysteic acid-functionalized alumina membranes can separate oil emulsions from frac and produced water.

## Results and Discussion

A membrane average pore size of 0.22 μm was chosen since this is in the high end of the ultrafiltration range and low end of the microfiltration range. The membrane should reject oil emulsion, bacteria and particulates by size exclusion; however, sugars and other soluble organic compounds should not be rejected. The pore size is sufficiently small that fouling occurs rapidly with types of components in produced water.

The membranes were functionalized by reacting at 85 °C with cysteic acid in DI water. Reactions were initially carried out in a static reaction vessel; however, it was found that for more than one membrane it is easier to perform the reaction by flowing the cysteic acid solution through the membrane. The treated membranes are very hygroscopic and will attain a wet appearance, even after being dried, even if they are stored in a low humidity environment. The surface area of the membrane (0.358 m^2^/g) is unchanged upon surface functionalization.

SEM analysis of the surface of the functionalized membranes is indistinguishable from the untreated membrane, but the difference can be seen from EDS analysis. In the EDS spectrum both sulphur and nitrogen peaks are observed for the functionalized membrane (Fig. 1e,f), which are absent for the un-functionalized membrane (Fig. 1d). Mapping of the functionalized membrane for nitrogen and sulfur showed even coverage across the entire cross section (Fig. 1h–l). Nitrogen and sulphur signals were not diminished after the membrane was washed repeatedly or after treatment of multiple batches of frac or produced water, confirming that the cysteic acid is covalently bound to the alumina surface^33^.

### Produced water analysis

The total carbon (TC), non-purgeable organic carbon (NPOC, also known as total organic content or TOC), and total inorganic carbon (TIC) for each produced water sample were measured (Table 1). In our previous report of the quantitative analysis of produced water samples, we found that the TOC of a shale produced water from wells recently drilled varies between 3 and 58 g/L^9^. Some of the samples studied herein are from dry gas wells and thus represent examples of water with lower initial carbon content. The NPOC is significantly higher than the TIC for most of the samples; however, both Roosevelt (Utah) and Scott Sugg (Texas) samples show a TIC:NPOC ratio >1, i.e., the carbonate content is higher than the organic content. There is no relationship between TIC and NPOC, which is consistent with the variation of geology between various shale reservoirs. The important point from this observation is that the source waters vary considerably and any process for treatment will have to deal with a range of feed compositions.

**Table 1:** TC, NPOC, and IC content (mg/L) of feed, concentrate, and permeate water for various frack flowback and produced water purified using a cysteic acid functionalized 0.22 μm α-alumina membrane.

Source	Sample	TC	NPOC	TIC
Marcellus	Feed	3808	3728	1460
	Concentrate	7468	6737	2410
	Permeate	108	83	25
Barnett	Feed	58550	43550	15000
	Concentrate	160900	120950	39950
	Permeate	838	638	200
Eagle Ford	Feed	9285	6095	3190
	Concentrate	25578	11197	14381
	Permeate	1050	1023	27
Scott Sugg	Feed	12056	376	11680
	Concentrate	14048	5204	8844
	Permeate	4104	248	3856
Humble	Feed	898	872	25
	Concentrate	1164	938	225
	Permeate	51.5	47.8	3.7
Roosevelt	Feed	6028	356	5672
	Concentrate	4828	328	4500
	Permeate	1.5	1.2	0.3

### Filtration analysis

**Figure 2:**
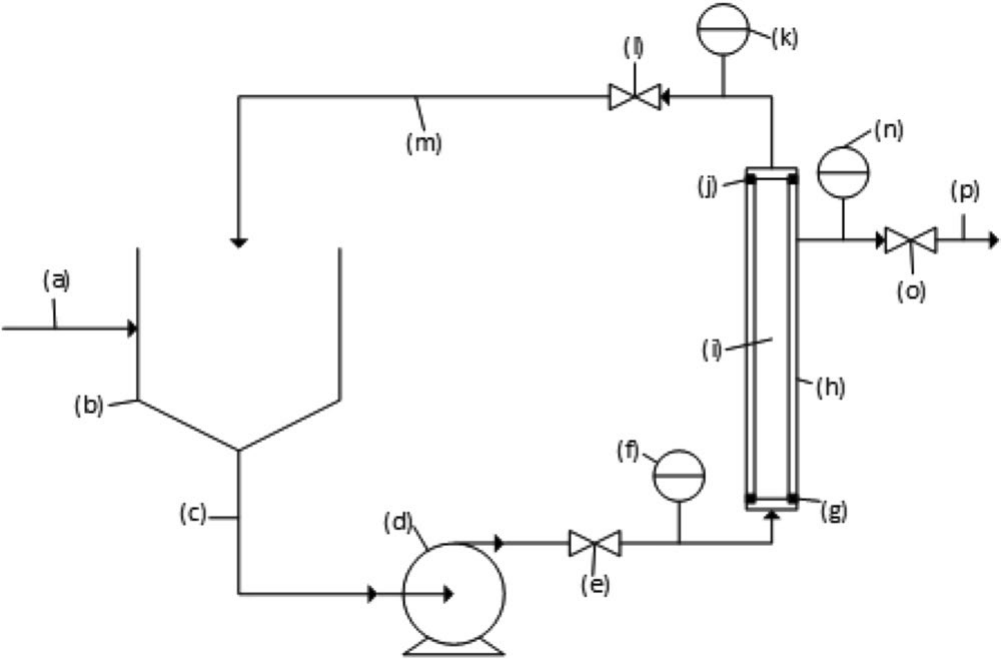
Schematic representation of membrane filtration unit with (**a**) feed inlet, (**b**) recirculation tank, (**c**) pump suction line (**d**) pump, (**e**) pump discharge valve, (**f**) pressure gauge pre-filter, (**g**) gasket holding membrane in housing, (**h**) membrane housing, (**i**) ceramic membrane, (**j**) gasket holding membrane in housing, (**k**) pressure gauge post-filter, (**l**) concentrate return line valve used to vary trans-membrane pressure, (**m**) concentrate return line, (**n**) permeate pressure gauge, (**o**) permeate control valve, (**p**) permeate line.

**Figure 3:**
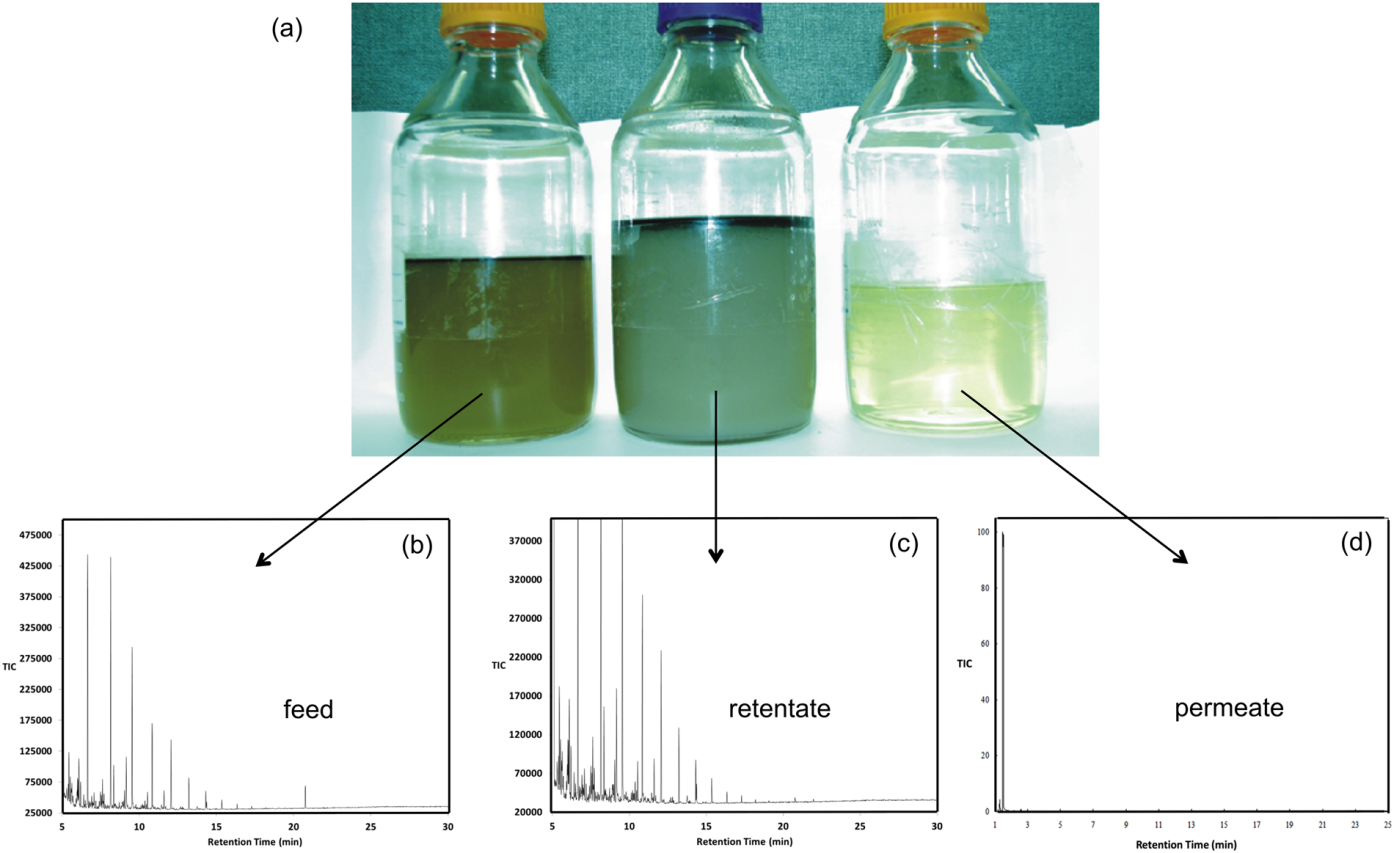
(**a**) Visual inspection of Marcellus shale produced water sample before and after filtration showing the feed (left), concentrate (center), and permeate (right) samples. Representative GC traces for Marcellus produced water using CHCl_3_ extraction for (**b**) feed, (**c**) retentate, and (**d**) permeate. The large peak in the permeate trace is due to the CHCl_3_ reference.

Qualitative and quantitative analyses were undertaken using the setup described in the Fig. 2. Initial analysis by inspection clearly demonstrated the ability of the membrane to screen colloidal hydrocarbons. The clarity of the permeate sample compared to the feed and the concentrated samples indicated that the membrane successfully removes large amounts of water contaminate (Fig. 3a). Analysis of the carbon content of both permeate and concentrate samples in comparison to the feed sample showed that there was significant removal of carbon material in the production water. The carbon of the permeate sample was in the low ppm range (Table 1). The same extremely low carbon content was evident for all types of water analysed. It is worth noting that while only the Roosevelt produced water comes close to being within EPA limits (0.01–1.0 mg/L) after purification, all of the samples are cleaned sufficiently for re-use in a hydraulic fracture or a water flood, allowing for re-use.

The percentage retention of carbon content by the membrane (a measure of the ability of the membrane to remove carbon from the permeate) was calculated using R = 1−(C_p_/C_r_), where R = rejection coefficient, C_p_ = permeate concentration and C_r_ = retentate concentration. For TC and NPOC the rejection coefficient was generally in the range 0.95–0.99 for produced water samples (Table 2). The Scott Sugg flowback sample shows the lowest rejection coefficients. The carbonate concentration (IC) in the feed is higher than the organic content (NPOC); however, NPOC in the permeate is reduced compared to the feed. The sample taken from the evaporation pit also showed a slightly lower rejection coefficient for a single pass; however, analysis of several samples collected from different locations in the pit showed a variation from 0.78–0.92. The rejection coefficient for TIC (>90%) for the samples, except the Scott Sugg, which is one of the highest TIC:NPOC ratio materials, and the low rejection is possibly associated with the low level of organic present.

**Table 2:** Rejection coefficient (r) for total carbon (TC), non-purgeable organic carbon (NPOC), and total inorganic carbon (TIC) for various frack flowback and produced waters purified using a cysteic acid functionalized 0.22 μm α-alumina membrane.

Source	TC	NPOC	TIC
Marcellus	0.985	0.987	0.989
Barnett	0.995	0.995	0.995
Eagle Ford	0.959	0.909	0.998
Scott Sugg	0.708	0.952	0.564
Humble	0.956	0.949	0.984
Roosevelt	0.999	0.996	0.999

A chemical analysis of the feed, concentrate and permeate water was undertaken using GC-MS analysis^9^. The GC traces for Marcellus produced water feed (Fig. 3b) and retentate (Fig. 3c) are almost identical, consistent with the NPOC results. In contrast, the trace for the permeate (Fig. 3d) shows only a few small peaks (in addition to the reference peak CHCl_3_ used for the solvent extraction^9^). The assignment for these peaks was based upon the fitting of the integrated mass spectrum for each peak. The detectable organic components that pass through the cysteic acid functionalized membrane are acetone, methylcyclohexane, and ethylcyclohexane. While acetone is miscible in water, the cyclohexanes are slightly soluble (16 and 3.8 mg/L, respectively), but below the NPOC measured in the permeate of the Marcellus shale produced water. Similar analysis of the permeate from the Barnett and Eagle Ford waters indicates that hydroxyacetonitrile, 1,1-dimethylcyclopropane, and 2-methyl-1-butene are potentially not rejected by the membrane. While hydroxyacetonitrile is highly soluble in water, the other two have very low solubility. These low molecular weight hydrocarbons have been observed in the permeate using un-functionalized membranes with a similar pore size as the membrane utilized here (0.22 µm).

**Figure 4:**
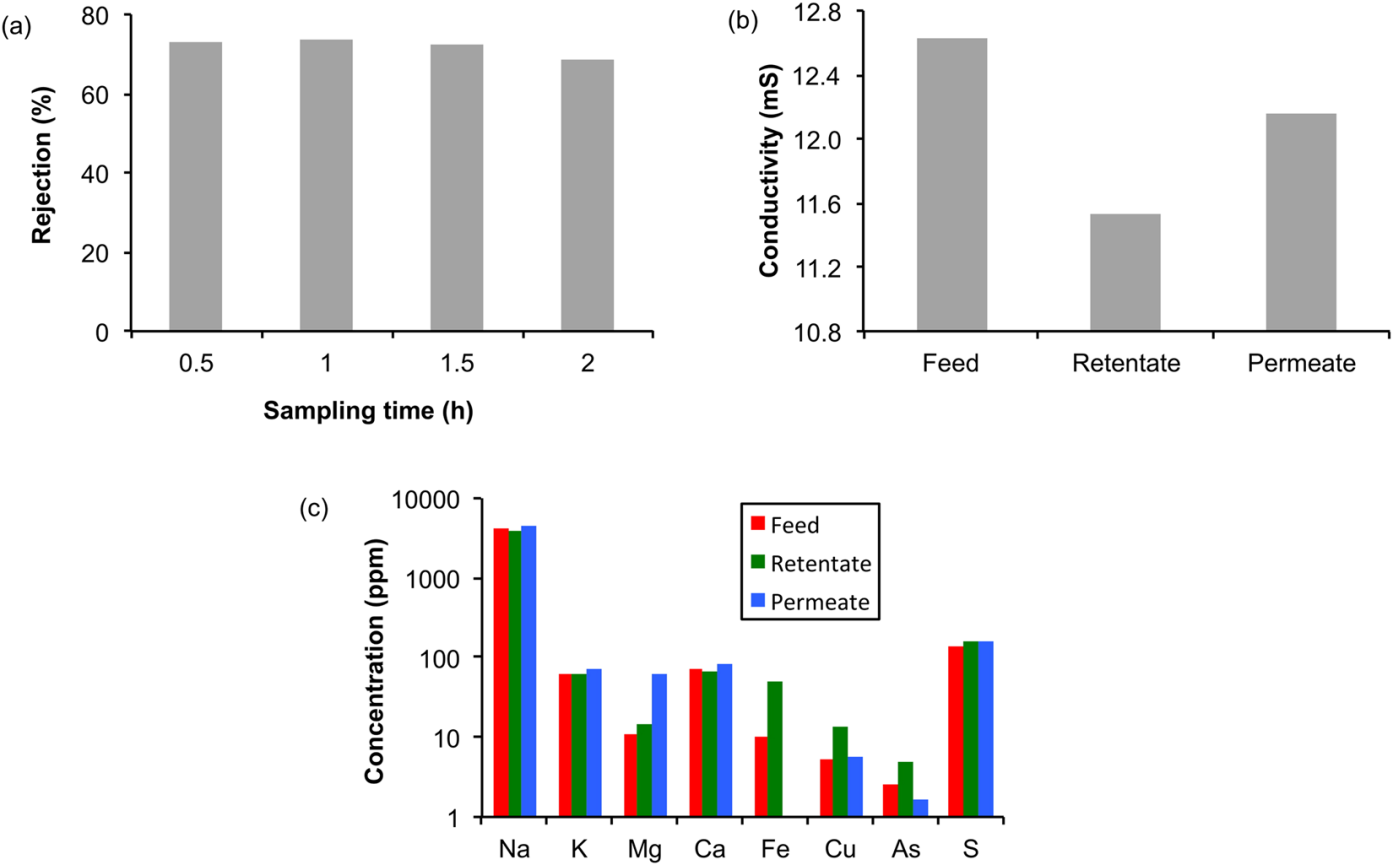
(**a**) Plot of percentage retention of guar gum in concentrate reconstructed from monomeric sugar, (**b**) conductivity (mS) of the feed, retentate and permeate for Marcellus produced water purified by a single pass through the cysteic acid functionalized 0.22 μm α-alumina membrane, and (**c**) ICP-OES analysis of high abundance metals in concentrate, feed and permeate water for Marcellus water.

The normal definition of flowback and produced is that the latter occurs once gas or oil have started to be produced. However, it is difficult to chemically differentiate the water. One potential differentiator is the presence of guar gum (or degradation components thereof). Gels of guar gum cross-linked with borax or a transition metal compounds are used in hydraulic fracturing fluids to provide viscosity^15,34^. The polysaccharide guaran (M_w_ ≈ 10^6^ Da) is the major component of guar gum. The ability to remove guar gum from flowback or produced water has proven difficult due to the higher amounts of TDS contained in water which contribute to membrane surface fouling. In addition, the solubility of hydrolyzed guar gum in water contributes to the difficulty in separating using microfiltration. However, this has proven to not be the case with our functionalized alumina membrane. For continual filtration (i.e., with return of both the concentrate and permeate to the feed) the rejection coefficient shows a slow decrease only after 2 hours of treatment (Fig. 4a).

The chemistry of a shale reservoir is unlike that of a conventional oil or gas reservoir that is flushed with transient water resulting in the equilibration of the rock and other components. The salt content of produced waters have been extensively reported^35^, and there is no direct relationship between the conductivity and pH indicating that the conductivity is a function of salt content and identity rather than acidity^9^. The conductivity of the retentate and permeate change slightly with respect to the feed as shown in Fig. 4b. Given the high organic rejection by the membrane, it is expected that the retentate have a lower conductivity, which was observed. Typical results for the inorganic components (determine by ICP-OES), shown in Fig. 4c, and remains more or less unaffected by the separation process except for iron. We believe iron retention is indicative of the membrane screening of bacterial colonies contained in the feed water, since the membrane does not reject soluble Fe^3+^. The reduction of iron is important since if the water is to be reused for hydraulic fracking then the iron concentration must be carefully controlled to inhibit premature cross-linking of gel based frack fluids^32^. Percentages of rarer metals are observed only in the concentrate but not in the feed or permeate, presumably as a result of retention of functionalized organic molecules that may ligate these metals, and should not be attributed to some form of reverse osmosis or ion exchange process.

The bacterial content of selected samples was determined before and after treatment with the cysteic acid functionalized 0.22 μm α-alumina membrane. Akob *et al*.^36^ has reported the typical microbiology of produced waters from Pennsylvania shale gas wells found that microbial viability were highly variable in the range of 66–9400 cells/mL. As seen in Table 3, the produced water shows bacteria counts in the region of 10^3^–10^6^ bacterial per mL (CFU/mL); however, as expected the removal is >99% because of the pore size not the functionalization.

**Table 3:** Measurement of colony-forming units per mL (CFU/mL) in selected frack flowback and produced waters before and after purification using a cysteic acid functionalized 0.22 μm α-alumina membrane.

Source	Feed (CFU/mL)	Permeate (CFU/mL)	% removal
Eagle Ford	8600	24	99.7
Scott Sugg	10^6^ (APB)	<10	99.9
Roosevelt	10^4^ (IB), 10^3^ (SRB)	0.0 (IB), 1.0 (SRB)	>99.9

### Membrane fouling and performance

**Figure 5:**
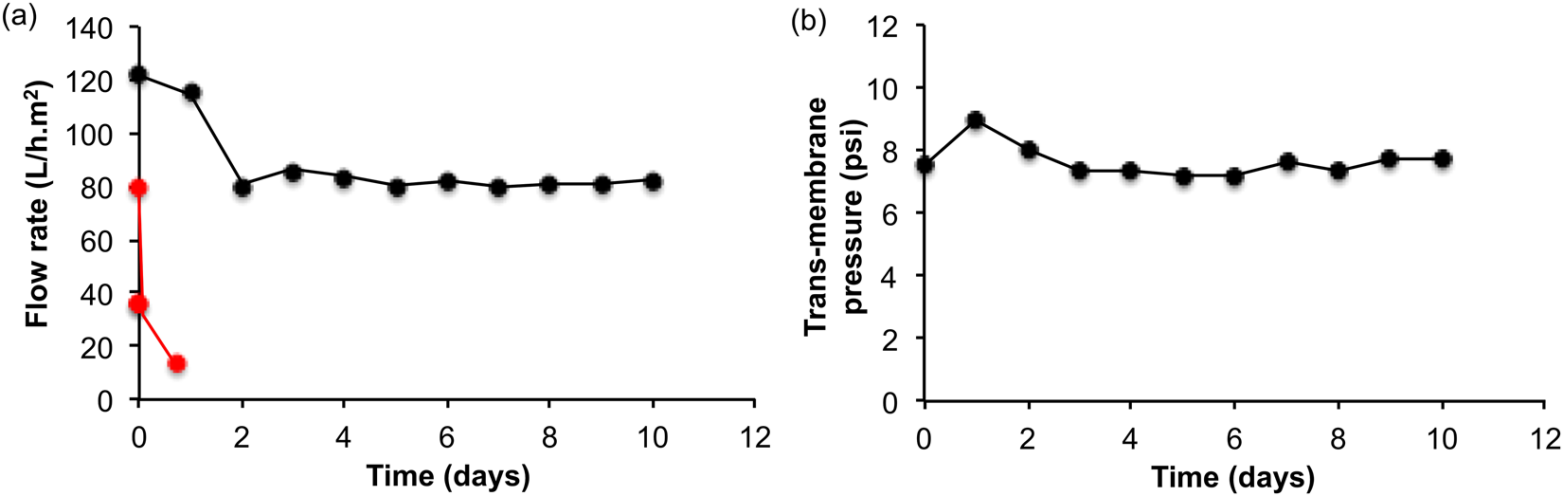
Plots of (**a**) permeate flux and (**b**) trans-membrane pressure over 10 days continual flow of cysteic acid functionalized 0.22 μm α-alumina membrane. For contrast, the permeate flux for an untreated membrane is shown in red.

One of the goals of using the cysteic acid functionalized super hydrophilic membrane was to minimize fouling and hence flux decline as compared to an un-functionalized membrane which suffers a decay on the permeate flow due to fouling from organics. Evolution of permeate flux rate (Qp) as a function of time (Fig. 5a) the permeate flux decreases initially but then stabilizes and remains so for the duration of the measurement. This is in complete contrast to analogous data for an unfunctionalized membrane that shows a dramatic decrease in permeate flux rate within 18 hours (Fig. 5a), under identical conditions. For the cysteic acid functionalized membranes the trans-membrane pressure (Fig. 5b) shows an initial build-up of pressure, which then subsides. The pressure then remains steady for the period of the measurement. This initial build up is a result of ‘saturation’ of the membrane with water and can be attributed to two factors. Firstly, due to the purging of the pores of air, which can block a pore and result in a lower area for permeate to pass through thus resulting in higher pressures and secondly, the establishment of a steady-state water layer (or layers) on the membrane surface. These water layers have been attributed to the reason for the extended lifetime of the functionalized membrane and its ability to avoid surface fouling. This means that the membranes can run longer and are more effective over a longer period of time.

The aligned orientation of the sulfonyl and amine moieties away from the membrane surface results in the membrane displaying significant hydrophilicity^27^. With the surface functionalization of the entire membrane the pore size has not been altered to any significant extent, i.e., the cysteic acid molecule is minuscule (ca. 9 Å) compared to the pore size of the membrane (2200 Å). The ability of the functionalized membrane to separate hydrocarbons from water when the hydrocarbon molecules have hydrodynamic diameters significantly smaller than the pore size of the membrane can be interpreted in several ways. Hydrophilic surfaces on membranes have been shown to be superior for the rejection of organic molecules as well as showing a reduction in membrane fouling^21–24^. This study has shown that the cysteic acid functionalization is not just on the exterior surface of the channels, but also on the internal surface of the pores as well (Fig. 1), and thus creates a highly ordered zwitter ionically charged super hydrophilic membrane surface, which repels the oil components when the quantities are sufficient to form globules (a second phase).

Separation of water and hydrocarbons is only one issue that the cysteic acid functionalization appears to address. Fouling of membranes is a complex phenomenon and involves the deposition of solute or particles onto the membrane surface or into the pores. Fouling is usually enhanced with high membrane surface roughness, increased feed concentration, increased membrane flux and is dependent on the nature of the membrane surface and solute interaction forces. Therefore, efforts have been made to create highly uniform membrane surfaces to limit fouling^21–24^. The low fouling of the cysteic acid functionalized alumina membrane can be related to the zwitter ionic nature of the surface creating a super hydrophilic layer that is wetted with water. When organic globules contact this wetted layer they are repelled and do not have access to the membrane surface in order for deposition to occur. The modified surface of the membrane significantly increases the membrane flux (e.g., 1130 L/h.m^2^ @ 1 bar DI water) when compared to the original membrane (504 L/h.m^2^ @ 1 bar DI water). The flux obtained at low pressure (<2 bar) for the modified membrane is the same magnitude for the untreated membrane operating at >4 bar. As the flux is the same, there will be no impact on fouling. However, the lower operating pressure means reduced operational cost as the pump needed is smaller and consumes less electricity. Each of these factors has a corresponding environmental benefit and could be quantified by a reduced carbon footprint.

## Conclusions

The present work shows that it is possible to treat frack flowback and produced water from shale oil and gas wells to reduce the hydrocarbon content to levels that are acceptable for re-use using a single pass through the membrane without pretreatment^37^. The high performance of this large pore size functionalized membrane with regards to hydrocarbon screening, high flux, low operating pressures, anti-fouling properties as well as ease of synthesis point to a new generation of hybrid inorganic membranes. The cysteic acid functionalization alters the properties of the membrane on the macro scale. The ability of this membrane to screen hydrocarbons of hydrodynamic diameters orders of magnitude smaller than the pore size appears a function of the solubility coefficient of the hydrocarbons and the resulting emulsified droplet rather than being attributed to direct separation as would result from a nano filtration membrane^38^.

## Methods

### Materials

All chemicals were purchased in high purity form from Sigma-Aldrich (Reference) and were used without further purification. Alumina membranes (0.22 μm nominal pore size) were purchased from Pall Corporation (Reference) and washed with DI water prior to treatment. All water used was purified via Millipore filtration to 18 MΩ deionized (DI). ICP standards (IV-ICPMS-71A) were obtained from Inorganic Ventures (Reference). Whatmann filters No. 40 were obtained from Fisher Scientific (Reference). Thermo Scientific 50 mL and 15 mL sterilized graduated conical centrifuge tubes were obtained from Fischer Scientific. Frac flowback and produced water samples were drawn from wells in the Marcellus (Pennsylvania), Eagle Ford (Texas), Barnett (New Mexico), Humble (Texas), Permian (Texas), Scott Sugg (Texas) and Roosevelt (Utah). Water samples were collected in 1-L mason jars. All jars used for sample collection were cleaned using detergent and rinsed thoroughly with DI water. The jars were then soaked in a base bath containing KOH in isopropyl alcohol for one week then rinsed thoroughly with ethanol then DI, followed by soaking in concentrated nitric acid for three days and again rinsed thoroughly with DI. All glassware used the same cleaning procedure. Samples were collected directly from frac tanks into which the produced water was directly piped. No mixing with other water sources was observed. All samples were collected without a headspace to reduce oxidation of the sample. All samples were stored and transported in sealed containers under refrigeration. Blank samples were tested using DI water. These showed that no chemicals were leached from the O-rings in the jar or contamination from other sources during handling and storage.

### Membrane functionalization

The reaction of the alumina membrane with cysteic acid was performed using a variation of previously reported procedures^32^. The alumina membranes were placed in an airtight glass container. The container was filled with DI water and placed under vacuum until the membrane stopped effervescing. This ensured removal of air from the interstitial pores. Vacuum was then removed and the glass column was heated to 85 °C for 24 hrs. The deionized water was drained from the container. The membrane was covered in a 1 M aqueous solution of cysteic acid; again vacuum was placed on the container until the membrane stopped effervescing. The solution was brought to gentle reflux for 2 days. The membrane was then allowed to return to room temperature. The membrane was again covered with DI and pumped down until the membrane stopped effervescing and gently heated to 50 °C then allowed to completely drain. This process was repeated three times, or until the water displayed pH = 6.

### Membrane characterisation

Thermogravimetric analysis (TGA) was conducted on a TA Instruments Q-600 simultaneous TG/DTA instrument, under argon using platinum pans. Analysis was conducted on TA Instruments analysis software. Infrared spectroscopy (IR) of functionalized and un-functionalized membranes was analysed on a Nicolet FTIR Infrared Microscope with diamond window. Bruaneaur-Emmet-Teller (BET) analysis was performed using the Quantachrome Autosorb-3B Surface Analyser. Samples were broken from the membrane and heated under vacuum at 80 °C for 24 hrs to remove any excess water. The samples were then purged with helium and analysed at 77 K under nitrogen. Scanning electron microscopy (SEM) analysis was conducted on FEI Quanta 400 a multiple stage high resolution field emission environmental scanning electron microscope (ESEM), both in scanning electron (SE) mode and energy dispersive X-ray scattering (EDS) mode, analysis was undertaken where stated either in Hi-Vac or Low-Vac mode. An acceleration voltage of 30 keV was used as well as a spot size of 4.0 to ensure a dwell time of approximately 30% for the EDS detector and to reduce charging. Samples were immobilized on an aluminium stub with carbon tape.

### Produced water treatment

Filtration experiments were conducted on a single pass-closed loop batch system. The membranes were subjected to filtration of produced water. The flow rates of permeate and concentrate as well as assembly pressures and substituent temperatures were monitored. Sampling of the original production water, permeate, and the concentrate were taken at specified times. These samples were analysed for carbon content, conductivity, elemental composition, and finally molecular composition.

### Water analysis

Bacteriological analyses were performed by Lance Energy Services, Inc. (Houston, TX). The water sample jars were decanted and pre-filtered three times with Whatmann Filter No. 40 before analysis to remove any samples containing visible particulate matter or non-dissolved matter. The filtered water was added to a glass 20 mL scintillation vial. Conductivity was measured on a calibrated pH/CON 510 Oakton analyser (WD-35610–10) using the protocol described previously^4^. Metal content of the produced and filtered water was analysed using ICP-OES (Inductively Coupled Plasma Optical Emission Spectrometer) carried out using an Optima 4300 DV spectrophotometer analyser with an AS-93 + auto sampler^9^. Carbon analysis was analysed using a Shimadzu TOC analyser (TOC-VCSH) using an auto-sampler (ASi-V) in TC and NPOC mode. Gas chromatography/mass spectroscopy (GC/MS) analyses were performed on an Agilent Technologies 5973 network mass selective detector with a quadrupole mass spectrometer with an Agilent Technologies 5973 network GC system, using a 30-m DB-1 capillary column (0.25-mm I.D., 0.25-μm film) and helium as the carrier gas. All samples were injected using an Agilent 7693 A Automatic Liquid Sampler with split/splitless injections of 1.0 μL, injection temperature was set at 285 °C.
